# The Prognostic Value and Perioperative Dynamics of the HALP Score in Placenta Accreta Spectrum Surgeries

**DOI:** 10.3390/jcm14217781

**Published:** 2025-11-02

**Authors:** Tuğçe Arslanoğlu, Sezin Uludağ, Oğuzhan Yürük, Hale Çetin Arslan, Pakize Özge Karkin, Seda Atak, Nuran Tamtürk, Serap Adıyaman, Deniz Kanber Açar, Alev Atış Aydın

**Affiliations:** 1 Clinic of Perinatology, Kanuni Sultan Süleyman Training and Research Hospital, Istanbul 34303, Turkey; 2 Clinic of Obstetrics and Gynecology, Kanuni Sultan Süleyman Training and Research Hospital, Istanbul 34303, Turkey

**Keywords:** HALP score, placenta accreta spectrum, preoperative risk stratification, surgical complications, cesarean hysterectomy

## Abstract

**Objective**: We aimed to evaluate the prognostic value of the hemoglobin, albumin, lymphocyte, and platelet (HALP) score in placenta accreta spectrum (PAS) surgeries and its perioperative dynamics as a marker of surgical and neonatal outcomes. **Methods**: This retrospective cohort included 100 patients with histopathologically confirmed PAS who underwent cesarean hysterectomy (2016–2025). The HALP was calculated within 24 h before delivery and reassessed at 6 and 24 h after delivery. Demographic, surgical, and neonatal variables were recorded. The primary outcome was the association between preoperative HALP and surgical morbidity; the secondary outcomes were perioperative HALP changes and neonatal correlations. ROC analysis identified cutoff values; multivariable regression was used to determine predictors of HALP variability. Internal validity was assessed via bootstrap resampling (1000 and 5000 iterations). **Results**: Preoperative HALP was significantly greater in patients with complications (24.14 vs. 22.58; *p* = 0.023). ROC analysis yielded a cutoff of 29.23, with 53.2% sensitivity and 82.0% specificity (AUC: 0.602, 95% CI: 0.51–0.69;). HALP showed a biphasic perioperative pattern, increasing at 6 h and then decreasing at 24 h (*p* < 0.001). Elevated HALP was independently associated with earlier gestational age at diagnosis, lower birthweight, and reduced Apgar scores. Bootstrap analyses revealed a stable AUC (~0.60) and consistent cutoff estimates across resamples. **Conclusions**: Higher HALP scores, which are typically markers of favorable nutritional status, are paradoxically linked to increased maternal morbidity and adverse neonatal outcomes in patients with PAS. HALP may, therefore, reflect placental invasiveness rather than maternal reserve. Its low cost and dynamic behavior highlight its potential utility in preoperative risk stratification for high-risk obstetrics.

## 1. Introduction

Placenta accreta spectrum (PAS) is a serious obstetric disorder caused by partial or complete loss of the decidua basalis, leading to trophoblastic invasion into the myometrium. The incidence of placenta previa has increased significantly with increasing cesarean section rates, particularly when placenta previa coexists [1]. PAS is associated with massive intrapartum bleeding, transfusion requirements, and surgical complications. Most cases necessitate planned hysterectomy and multidisciplinary care. The average blood loss often exceeds 3000 mL, with many patients requiring intensive care support [2]. In this high-risk setting, objective preoperative assessment of systemic status is crucial for clinical management.

In recent years, interest in identifying reliable biomarkers that can stratify the severity of PAS and predict adverse outcomes has increased. While ultrasonography and magnetic resonance imaging (MRI) remain the cornerstones of antenatal diagnosis, their ability to predict perioperative outcomes is limited. Consequently, laboratory indices reflecting systemic inflammation and nutritional reserves have gained attention as potential complementary tools that may enhance risk assessment beyond imaging findings.

Recently, several hematologic indices commonly used in oncologic surgery have been shown to predict clinical prognosis by reflecting systemic inflammation, the immune response, and nutritional status. The HALP score, a composite of hemoglobin, albumin, lymphocyte, and platelet values, was first studied in oncology and has gained interest in obstetrics. Studies suggest that low earlypregnancy HALP scores may be associated with complications such as preeclampsia, fetal growth restriction, and gestational diabetes [3,4].

The PAS not only reflects the degree of placental invasion but also affects systemic inflammation and physiological reserves. Increased inflammatory activity in advanced cases may lead to hematologic disturbances, including anemia, hypoproteinemia, lymphopenia, and thrombocytopenia, which directly influence the HALP score [5]. Additionally, intraoperative blood loss and systemic stress may significantly reduce postoperative HALP levels [6]. Thus, the HALP score may serve a dual role: in predicting preoperative risk and tracking postoperative complications.

The HALP score offers a more comprehensive evaluation by simultaneously integrating immune, hematologic, and nutritional indicators. In this respect, HALP may capture both baseline maternal reserves and the dynamic physiological response to surgical stress within a single index.

Hematologic markers such as the neutrophil/lymphocyte ratio (NLR), platelet/lymphocyte ratio (PLR), and systemic inflammation response index (SIRI) have shown diagnostic value in PAS [7]. However, the HALP score offers a more comprehensive assessment by combining immune and nutritional indicators. Optimizing hematologic and nutritional parameters preoperatively in patients with low HALP scores may help prevent complications, whereas abrupt postoperative declines in HALP may signal emerging complications. Despite its potential, data on the HALP application in PASs remain limited. This study aims to address this gap.

## 2. Materials and Methods

This retrospective descriptive study was conducted using archival records from the Perinatology Clinic of Obstetrics and Gynecology, Kanuni Sultan Süleyman Training and Research Hospital, University of Health Sciences. All pregnant women who were diagnosed with placenta accreta spectrum (PAS) preoperatively between January 2016 and January 2025, who were confirmed intraoperatively, who were treated with hysterectomy, and whose medical records were complete were included. A total of 100 PAS cases were evaluated. Patients with incomplete laboratory data, multiple gestations, major hematologic or hepatic comorbidities, or concurrent malignancies were excluded. Data, including patient files, operative notes, and laboratory results, were collected retrospectively from the hospital’s automated system. Given the rarity of PAS surgeries and their concentration in referral units, this single-center retrospective design ensured the homogeneity of surgical management and complete data capture. To address concerns related to the modest sample size, we emphasized effect sizes with 95% confidence intervals and additionally performed bootstrap resampling (1000 and 5000 iterations) for key analyses.

Hematologic and biochemical parameters were obtained for both the preoperative and postoperative periods. The HALP score was calculated via the following formula:

- HALP = (Hemoglobin [g/L] × Albumin [g/L] × Lymphocyte count [/mm^3^]) ÷ Platelet count [/mm^3^].
- (Hemoglobin and albumin values were converted from g/dL to g/L by ×10; lymphocyte and platelet counts were recorded as 10^3^/µL, equivalent to/mm^3^. HALP is unitless).

The preoperative HALP score was calculated via laboratory data obtained within 24 h before delivery. Postoperative scores were measured at 6 and 24 h separately to monitor early physiologic response and systemic stress dynamics.

Demographic, obstetric, perinatal, surgical, and postoperative data were reviewed for each patient. Maternal age, gravida, parity, BMI, blood type, gestational age at PAS diagnosis, and gestational age at delivery were recorded. Clinical details included the urgency of surgery (elective/emergency), placental location (anterior, posterior, or previa), intraoperative complications (e.g., bladder, ureter, or bowel injury), and surgical procedures performed. Postoperative variables included hospital stay duration, ICU follow-up time, and erythrocyte transfusion volume. Neonatal outcomes included birthweight and Apgar scores at 1 and 5 min.

The primary aim was to assess the associations between preoperative HALP scores and intraoperative blood loss and transfusion requirements. The secondary aims included relationships with ICU need, surgical complications, postoperative HALP changes, and hospital stay. Thus, the potential of HALP as both a preoperative risk marker and a postoperative outcome predictor was evaluated.

This retrospective, noninterventional study was based on data from patient records. Ethical approval was obtained from the Clinical Research Ethics Committee of Istanbul Kanuni Sultan Süleyman Training and Research Hospital (Decision No. 2025–102). All the data were anonymized in accordance with the Declaration of Helsinki.

Statistical analysis was performed via an NCSS 2007 (Kaysville, UT, USA). Descriptive statistics (mean, standard deviation, median, frequency, ratio, minimum, and maximum) are reported. Normality was assessed via the Shapiro–Wilk test. The Friedman and Wilcoxon tests were used for repeated measures; the Mann–Whitney U test was used for between-group comparisons. ROC analysis identified the optimal cutoff value, and linear regression was used to assess predictors. All tests were two-tailed, and significance was set at *p* < 0.01 and *p* < 0.05. Because the complication subgroup was relatively small, conventional inferential tests were complemented with bootstrap confidence intervals (1000 resamples) for ROC analysis and regression models to examine the stability of the estimates. No a priori sample size calculation was feasible; therefore, the precision of the estimates was prioritized over post hoc power calculations.

## 3. Results

A total of 100 patients who underwent surgery for PAS were included. The placental location was anterior in 23% and posterior in 77% of the cases. Surgical complications occurred in 12 patients (12%), including 10 with bladder injuries, 1 with uterine rupture, and 1 with relaparotomy. Twenty-three patients (23%) were elective, whereas 77% were emergent. The demographic findings are summarized in Table 1.

The perioperative hematologic and immunonutritional parameters were analyzed in detail. Hemoglobin, albumin, lymphocyte, and platelet levels, along with derived HALP scores, were assessed preoperatively and at 6 and 24 h post-operatively. The mean values and temporal changes are summarized in Table 2.

**Figure 1:**
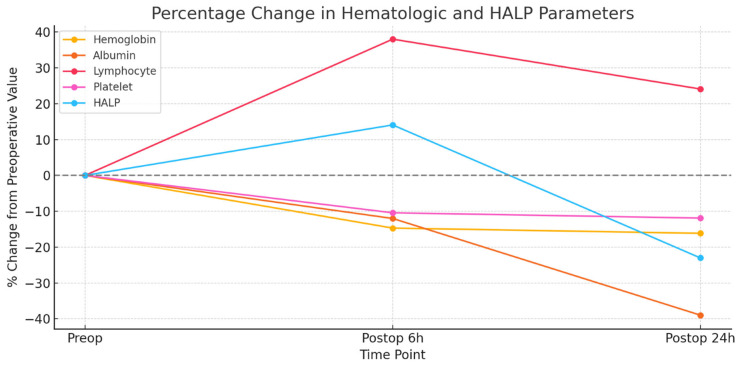
Percentage changes in hematological parameters and HALP scores at various perioperative time points (preoperative, postoperative, 6th hour, and 24th hour) in patients with placenta accreta. The values are expressed relative to the preoperative baselines. ALB and Hb levels progressively decreased, whereas HALP scores increased at 6 h but markedly decreased by 24 h.

Hematologic and biochemical parameters were compared preoperatively and at 6 and 24 h post-operatively via the Friedman test (*p* < 0.01). The mean hemoglobin levels were 10.96 ± 0.97, 9.35 ± 1.6, and 9.19 ± 1.65, respectively, indicating a significant difference overall (*p* = 0.001), although the change from 6 to 24 h was not significant (*p* = 0.240). Albumin levels decreased progressively from 4.08 ± 0.56 to 3.59 ± 0.54 and 2.49 ± 0.43, with all comparisons being significant (*p* = 0.001). The lymphocyte counts were 10 ± 3.51, 13.8 ± 5.34, and 12.41 ± 3.9; the differences among all time points were significant (*p* = 0.001), including at the 6th to 24th hours (*p* = 0.006). The platelet counts decreased from 214.08 ± 59.2 to 191.76 ± 56.15 and 188.6 ± 58.88, with a significant overall change (*p* = 0.001), although there was no significant difference between the last two time points (*p* = 0.708). The HALP score increased from 21.81 ± 8.99 preoperatively to 24.88 ± 10.97 at 6 h and then decreased to 16.79 ± 10.62 at 24 h, and these changes were statistically significant (*p* = 0.001). The perioperative temporal changes in hematological parameters and HALP scores are illustrated in Figure 1. This biphasic pattern is biologically plausible. The transient rise at 6 h may reflect perioperative fluid shifts, hemodilution, and acute inflammatory mobilization of lymphocytes following major surgery, whereas the subsequent decline by 24 h likely results from cumulative blood loss and progressive decreases in hemoglobin and albumin. These mechanisms highlight HALP as a dynamic marker that reflects evolving physiological stress rather than a static immunonutritional index.

The clinical and obstetric parameters of patients with and without complications, including maternal characteristics, surgical timing, transfusion requirements, neonatal outcomes, and postoperative recovery metrics, were compared. The detailed results are summarized in Table 3.

The preoperative HALP score was significantly greater in patients with complications than in those without complications (24.14 ± 7.36 vs. 22.58 ± 10.53; *p* = 0.023). Conversely, at the 6th postoperative hour, HALP scores were significantly lower in the complication group (21.74 ± 10.48 vs. 23.8 ± 10.38; *p* = 0.013). No statistically significant difference was observed in the 24 h post-operative HALP scores between the groups (*p* = 0.065). Detailed comparisons are presented in Table 4.

As shown in Table 5, multiple linear regression revealed a significant model for predicting preoperative HALP values (F = 26.632, *p* < 0.001). A moderately strong positive correlation was found between the predictors and the HALP score (R = 0.712), with the model explaining 50.7% of the variance (*p* < 0.01). Among the predictors, gestational age at diagnosis (β = –0.603), neonatal birth weight (β = –0.005), 1 min Apgar (β = –1.799), and 5 min Apgar (β = –2.588) were significantly and negatively associated with preoperative HALP values (all *p* < 0.001).

In conclusion, elevated preoperative HALP scores were significantly associated with earlier gestational age at diagnosis, lower birthweight, and reduced Apgar scores at both 1 and 5 min.

**Figure 2:**
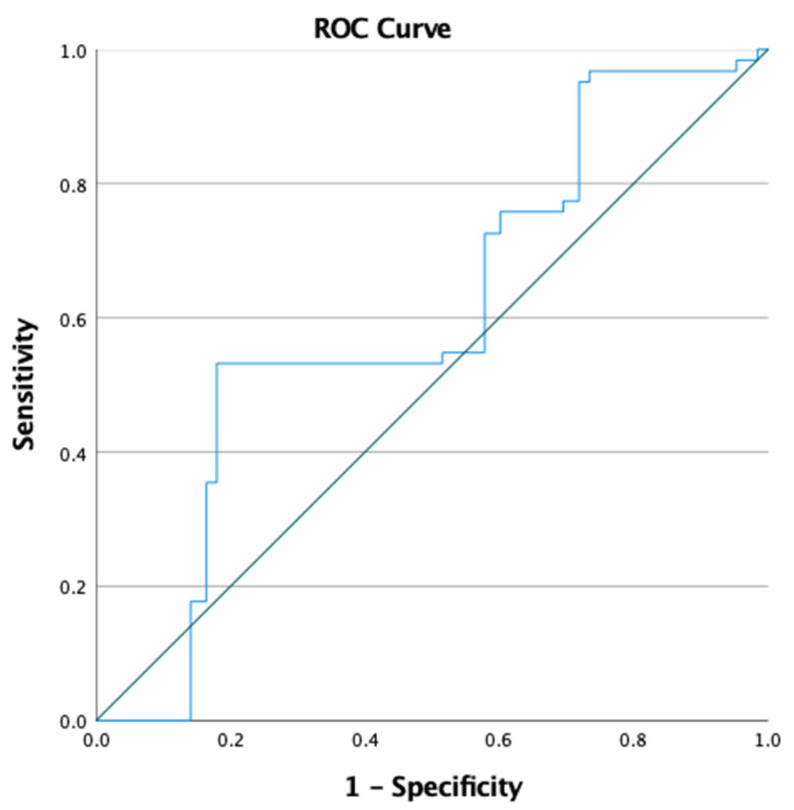
Receiver operating characteristic (ROC) curve of the preoperative HALP score for predicting surgical complications in the placenta accreta spectrum. The optimal cutoff value was 29.23, yielding an AUC of 0.602 (95% CI: 0.51–0.69), with 53.2% sensitivity and 82.0% specificity.

As shown in Table 6, a preoperative HALP cutoff of 29.23 was used to predict complications, with 53.2% sensitivity and 82% specificity. Figure 2. ROC curve showing the value of the preoperative HALP score for the prediction of complications.

### Internal Validation (Bootstrap Analysis)

In the 1000-bootstrap analysis with the cutoff fixed at 29.23, the mean AUC was 0.598 (95% CI 0.400–0.778), the sensitivity was 0.75 (95% CI 0.46–1.00), and the specificity was 0.15 (95% CI 0.08–0.23). In the 5000 bootstrap analysis with the optimal cutoff re-estimated at each iteration, the mean AUC was 0.597 (95% CI 0.402–0.780), the average optimal cutoff was 18.29 (95% CI 11.78–31.89), the sensitivity was 0.71 (95% CI 0.29–1.00), and the specificity was 0.63 (95% CI 0.14–0.93). These results indicate modest but consistent discrimination with stable estimates across resamples. The bootstrap confidence intervals were highly consistent with those of the primary analyses, supporting the robustness of the observed associations despite the limited sample size. The bootstrap confidence intervals were highly consistent with those of the primary analyses, supporting the robustness of the observed associations despite the limited sample size, as summarized in Table 7. Importantly, while the discriminatory power of HALP remains modest (AUC ≈ 0.60), the stability of the cutoff across resampling suggests that HALP may still hold value as a complementary biomarker when integrated with other established clinical parameters in decision-making processes.

## 4. Discussion

In this study, the prognostic value of the preoperative HALP score was evaluated in cases of placenta accreta spectrum (PAS), and a comparative analysis was performed against existing biomarker paradigms in the literature. According to our findings, patients with higher preoperative HALP scores had a significantly increased rate of complications (24.14 vs. 22.58; *p* = 0.023). Moreover, elevated HALP scores were negatively correlated with earlier gestational age at diagnosis, lower birth weight, and lower Apgar scores. These findings suggest that, in contrast to the conventional paradigm of “low HALP = poor prognosis,” the HALP score may offer a distinct and context-specific prognostic profile in obstetric settings. However, the absence of a significant difference between the complication and non-complication groups at 24 h indicates that the discriminative capacity of HALP may be time dependent, with greater utility in the preoperative and early postoperative windows but limited long-term predictive value.

Previous studies have demonstrated that lower HALP scores are associated with increased mortality and morbidity in fields such as oncology and cardiovascular surgery [8,9]. In the obstetric literature, however, HALP has been linked primarily to inflammatory conditions such as preeclampsia and hyperemesis gravidarum [4,10]. For example, in preeclampsia, lower HALP scores have been reported to correlate with poorer maternal and fetal outcomes, whereas our study revealed that higher HALP scores were predictive of PAS-related complications [3]. These findings suggest that the HALP score may exhibit distinct behavior in invasive placental pathologies such as PAS.

The association between elevated HALP scores and complications in PAS patients may reflect a distinct pathophysiological mechanism whereby trophoblastic invasion alters hematologic and immunologic profiles. In deeply invasive cases, the absence of overt antenatal anemia or inflammation may lead to relatively high preoperative hemoglobin, albumin, and lymphocyte levels [11]. While this results in elevated HALP scores preoperatively, a significant decline postoperatively suggests that dynamic changes may provide greater prognostic insight than a single measurement.

At a cutoff of 29.23, the preoperative HALP score predicted complications with high specificity (82%), supporting its potential role in enhancing multidisciplinary preparedness. However, its modest AUC of 0.602 underscores that HALP alone has limited discriminatory capacity and cannot serve as a stand-alone prognostic tool. This degree of accuracy is nevertheless comparable to that of several individual hematologic and inflammatory markers previously explored in obstetric and surgical risk prediction. Importantly, the HALP score integrates four routinely measured laboratory parameters into a composite index that reflects the interplay between hematologic reserves, nutritional status, the immune response, and coagulation balance. In this context, HALP may provide insights that are pathophysiologically distinct from those offered by single parameters such as CRP, fibrinogen, or neutrophil counts or even from derived ratios such as the systemic inflammatory response index (SIRI) or the delta neutrophil index (DNI) [7]. Its ease of calculation and negligible cost make it an attractive adjunct in real-world clinical settings, particularly where advanced biomarkers or molecular assays are unavailable. From a translational perspective, the integration of HALP into multiparametric risk models—alongside ultrasound or MRI-based PAS scoring systems and established laboratory markers—could enhance preoperative counseling, surgical planning, and perioperative triage. Therefore, while the prognostic strength of the preoperative HALP score is modest in isolation, its true clinical value may emerge when it is applied as a complementary component within broader, multimodal decision-support frameworks for PAS management.

This study highlights three potential clinical applications of the HALP score. First, the decline observed at 6 h post-operatively may help identify high-risk patients early, enabling timely interventions such as transfusions or ICU admission. Second, with the increasing incidence of PAS due to the increased number of cesarean deliveries [12], the HALP score may serve as a practical triage tool—particularly in resource-limited settings. Third, combining HALP with imaging modalities (e.g., MRI) could enhance existing PAS scoring systems by incorporating physiologic markers into surgical planning. These findings suggest that the HALP score may evolve into a pathophysiology-based clinical decision support tool for PAS management.

Preoperative HALP scores greater than 29.23 were found to be associated with increased surgical morbidity, whereas the decrease observed at postoperative hour 6 may represent early biological signs of acute complications. Accordingly, the HALP score emerges not only as a retrospective indicator but also as a dynamic monitoring tool that can inform real-time clinical decision-making. Within this framework, interventions such as targeted transfusion strategies and intensive care planning may be implemented more proactively on the basis of HALP-guided risk stratification. In an era marked by a global rise in cesarean delivery rates, the use of low-cost and widely accessible parameters such as the HALP score may hold strategic value for predicting complication risk, particularly in resource-constrained healthcare settings [13].

In our study, the HALP score increased from a preoperative mean of 21.81 ± 8.99 to 24.88 ± 10.97 at postoperative hour 6 and then decreased to 16.79 ± 10.62 at hour 24, indicating a statistically significant difference across all time points (*p* = 0.001). This biphasic trend underscores the dynamic nature of the HALP score. Given that the score is calculated as hemoglobin × albumin × lymphocyte/platelet, a transient increase in lymphocyte count and a postoperative decrease in platelet count—despite decreases in hemoglobin and albumin—may explain the paradoxical increase observed at hour 6. This early postoperative rise likely reflects immune mobilization and compensatory hematologic responses triggered by acute surgical stress. By hour 24, a further decrease in hemoglobin and albumin levels, combined with a decrease in lymphocyte count, resulted in a significant decrease in the HALP score. The time-dependent fluctuations of each component suggest that HALP functions not merely as a static measurement but rather as a composite reflection of evolving pathophysiological processes. Therefore, serial assessments of HALP may provide more meaningful clinical insight than isolated measurements do [14]. The observed biphasic pattern is also likely to be influenced by perioperative factors. Intraoperative blood loss, fluid resuscitation, and transfusion practices may have contributed to the transient rise at 6 h and subsequent decline at 24 h. While our retrospective dataset did not allow detailed adjustment for these variables, their potential confounding role should be acknowledged and warrants investigation in future prospective studies.

Our multiple linear regression analysis revealed significant and inverse associations between the preoperative HALP score and key clinical parameters. Specifically, gestational age at diagnosis (β = –0.603), birth weight (β = –0.005), and 1- and 5 min Apgar scores (β = –1.799 and –2.588, respectively) were significantly correlated with higher HALP scores (*p* < 0.001 for all). These findings suggest that elevated preoperative HALP values are associated with earlier diagnosis, lower birth weight, and worse neonatal status. From a clinical perspective, these associations suggest that patients with elevated preoperative HALP scores may represent a subgroup at risk for earlier PAS diagnosis, lower birthweight, and impaired neonatal adaptation. In practice, such cases may warrant intensified intrapartum preparedness (including early cross-matched blood availability) and proactive neonatal team involvement to optimize perinatal outcomes. Notably, HALP levels may also be influenced by baseline maternal comorbidities, nutritional deficiencies, or chronic inflammatory states, which could confound the associations observed. This further underscores the need for cautious interpretation and prospective validation.

This finding supports the paradoxical pattern observed in our study: a higher HALP is correlated with increased complication risk. In the context of PAS, elevated HALP may reflect more aggressive trophoblastic invasion and heightened immune activity. Thus, a high HALP score may not indicate better immunonutritional status but rather advanced placental pathology and increased fetal–maternal stress [15].

One of the most striking findings of our study was the significant association between elevated preoperative maternal HALP scores and adverse fetal outcomes—contrary to conventional expectations. Higher HALP values were associated with earlier PAS diagnosis, lower neonatal birth weights, and reduced Apgar scores at both 1 min and 5 min, indicative of compromised fetal growth and perinatal adaptation.

While prior studies, especially in preeclampsia, have linked low HALP scores to poor fetal outcomes, our results suggest the opposite. To explain this paradox, we propose a “reverse Warburg effect”: akin to malignant cells in cancer, invasive trophoblasts in the PAS may consume maternal metabolic resources to sustain their proliferation, thereby compromising fetal oxygenation and nutrient supply.

In this “metabolic sacrifice” model, a seemingly adequate maternal HALP score—typically reflecting preserved immunonutritional status—may in fact signal a placenta that disrupts maternal–fetal equilibrium to sustain its own invasive growth. Rather than indicating physiologic stability, elevated HALP may reflect pathologic placental dominance at the cost of fetal well-being. This observation highlights the necessity of interpreting obstetric biomarkers within their specific pathophysiological framework and calls into question the conventional assumption that higher scores universally predict better outcomes [16,17]. This interpretation remains hypothesis-generating but is supported by emerging evidence that trophoblasts undergo cancer-like metabolic reprogramming, with increased glycolytic activity and altered mitochondrial function to sustain proliferation, invasion, and adaptation under hypoxic stress. Recent studies on placental metabolism highlight glycolytic dominance and bioenergetic plasticity as central features of invasive trophoblast biology, which lends biological plausibility to our proposed framework [18,19].

Future research should aim to validate the prognostic utility of HALP in larger, prospective, multicenter cohorts and explore its integration into multimodal risk models combining HALP with established placental biomarkers such as sFlt-1/PlGF and angiogenic factor profiling [20,21]. Assessing how HALP dynamics respond to nutritional or perioperative optimization strategies may further clarify its modifiability and real-world clinical value. Such investigations are critical for determining whether HALP may evolve from a research metric to a reliable clinical decision-support tool in high-risk obstetric practice.

## 5. Limitations

This study has certain limitations. Foremost, its retrospective, single-center design limits external validity and may introduce selection bias. The relatively modest sample size, coupled with heterogeneity in the definition and severity of surgical complications, may constrain the generalizability of the findings. Furthermore, each component of the HALP score—hemoglobin, albumin, lymphocyte count, and platelet count—is susceptible to fluctuations due to extrinsic factors such as hydration status, infection, nutritional deficiencies, or comorbid hematologic conditions. In addition, perioperative factors such as intraoperative blood loss, fluid replacement, and transfusion practices may also have influenced the dynamic perioperative changes in HALP, and these factors could not be adjusted for in the present study. Consequently, HALP should be interpreted cautiously and used as an adjunct, rather than a stand-alone tool, within a comprehensive multidisciplinary risk assessment strategy.

## 6. Conclusions

This study demonstrated the paradoxical behavior of the HALP score in cases of placenta accreta spectrum (PAS), challenging traditional assumptions and suggesting previously unrecognized systemic mechanisms. Elevated preoperative HALP scores are not necessarily indicative of better maternal status but are instead associated with increased surgical morbidity and adverse neonatal outcomes.

In particular, the coexistence of a HALP score greater than 29 and a serum ALB level less than 2.5 g/dL was found to be correlated with higher complication rates and poorer fetal outcomes. These findings highlight the potential utility of the HALP score as a preoperative risk stratification tool in PAS patients. Additionally, the significant decrease observed at postoperative hour 6 may represent an early marker of acute physiological stress, providing a window for timely intervention.

Rather than serving as a static immunonutritional marker, the HALP score in PAS appears to function as a dynamic parameter reflecting the extent of trophoblastic invasion and maternal systemic burden. This study supports the integration of HALP into perioperative evaluation strategies and emphasizes the importance of interpreting such biomarkers within their disease-specific context. In practical terms, patients presenting with elevated preoperative HALP scores (>29) may benefit from closer perioperative surveillance, early preparation for blood transfusion, and consideration for postoperative intensive care monitoring. Moreover, the transient rise observed at 6 h post-operatively could serve as an early warning signal, prompting timely intervention before complications fully develop.

Although our study was derived from a single-center retrospective cohort, our bootstrap-supported results provide clinically relevant signals that warrant validation in larger, multicenter studies. Ultimately, our findings suggest that PAS should not be regarded merely as an anatomic or surgical condition but rather as a systemic disorder with quantifiable hematologic and metabolic components. Within this framework, the HALP score may serve as a low-cost, dynamic, and clinically meaningful adjunct to support both preoperative risk stratification and postoperative management in high-risk obstetric practice.

## Figures and Tables

**Table 1:** Baseline Maternal, Perinatal, and Surgical Characteristics.

	Mean ± Ss	Min–Max (Median)
Age (Years)	33.19 ± 4.38	23–44 (33)
Gravida	4.22 ± 1.75	2–14 (4)
Parity	2.79 ± 1.49	1–12 (3)
Number of Previous Cesarean Section	2.43 ± 1.07	1–6 (2)
Gestational Age at Diagnosis (week)	28.06 ± 5.85	15–38 (29)
Gestational Age at Surgery (week)	34.14 ± 2.8	23–38 (34)
Body Mass Index (kg/m^2^)	28.72 ± 4.43	19.31–38.08 (28.19)
Postoperative Use of Packed Red Blood Cells (UI)	2.42 ± 2.25	0–13 (2)
Length of ICU stay (days)	3.26 ± 1.66	2–7 (3)
Postoperative ward stay duration (days)	3.17 ± 1.18	2–9 (3)
Total Hospital Stay (days)	3.46 ± 1.75	2–12 (3)
Baby Weight (g)	2570.2 ± 583.73	510–3420 (2645)
Apgar 1st Min.	6.11 ± 1.79	0–9 (6)
Apgar 5th min.	8.14 ± 1.76	0–10 (9)
Day 1 Drain Volume (mL)	385.25 ± 271.15	100–1600 (300)

**Table 2:** Comparison of Measurements by Period.

Parameters	Preoporative Mean ± SD (Min–Max) (Median)	Postoperative 6 h Mean ± SD (Min–Max) (Median)	Postoperative 24 h Mean ± SD (Min–Max) (Median)	*p* (Friedman)	Preop vs. 6 h	Preop vs. 24 h	6 h vs. 24 h
Hemoglobin (g/dL)	10.96 ± 0.97 (8.9–14) (11)	9.35 ± 1.6 (5.5–13.6) (9.4)	9.19 ± 1.65 (4.4–14.4) (9.2)	0.001 **	0.001 **	0.001 **	0.240
Albumin (g/dL)	4.08 ± 0.56 (2.82–4.98) (4.04)	3.59 ± 0.54 (2.48–4.7) (3.53)	2.49 ± 0.43 (1.6–3.58) (2.49)	0.001 **	0.001 **	0.001 **	0.001 **
Lymphocyte (10^3^/µL)	10 ± 3.51 (4–27) (9)	13.8 ± 5.34 (3.4–29) (13)	12.41 ± 3.9 (3–26) (12.2)	0.001 **	0.001 **	0.001 **	0.006 **
Platelet (10^3^/µL)	214.08 ± 59.2 (111–359) (201)	191.76 ± 56.15 (84–328) (190)	188.6 ± 58.88 (35–364) (188)	0.001 **	0.001 **	0.001 **	0.708
HALP	21.81 ± 8.99 (5.15–66.44) (19.48)	24.88 ± 10.97 (9.78–61.08) (21.8)	16.79 ± 10.62 (3.17–90.85) (15.05)	0.001 **	0.001 **	0.001 **	0.001 **

*Friedman test* ** *p* < 0.01.

**Table 3:** Comparison of Measurements According to Complications.

		*n*	Mean ± Ss	Min–Max (Median)	*p*
Age(year)	Yes	12	34.58 ± 5.05	29–44 (34)	0.409
	No	88	33 ± 4.28	23–44 (32.5)	
Gravida	Yes	12	3.83 ± 1.64	2–7 (3.5)	0.373
	No	88	4.28 ± 1.77	2–14 (4)	
Parity	Yes	12	2.42 ± 1.24	1–4 (2.5)	0.406
	No	88	2.84 ± 1.52	1–12 (3)	
Number of Previous Cesarean Sections	Yes	12	2.42 ± 1.24	1–4 (2.5)	0.969
	No	88	2.44 ± 1.05	1–6 (2)	
Gestational Age at Diagnosis (week)	Yes	12	27.5 ± 5.78	18–36 (25.5)	0.629
	No	88	28.14 ± 5.89	15–38 (29)	
Gestational Age at Surgery (week)	Yes	12	32 ± 5.26	23–36 (34.5)	0.336
	No	88	34.44 ± 2.16	26–38 (34)	
Body Mass Index (kg/m^2^)	Yes	12	30.39 ± 5.58	20.46–37.25 (32.55)	0.203
	No	88	28.49 ± 4.24	19.31–38.08 (28.02)	
Postoperative Use of Packed Red Blood Cells (UI)	Yes	12	4.5 ± 3.56	0–13 (3)	0.014 *
	No	88	2.14 ± 1.87	0–8 (2)	
Length of Stay in Intensive Care Unit (day)	Yes	12	3.67 ± 2.19	2–7 (2.5)	0.843
	No	88	3.2 ± 1.58	2–7 (3)	
Postoperative Service Duration(day)	Yes	12	3.92 ± 1.88	2–9 (3.5)	0.087
	No	88	3.07 ± 1.03	2–6 (3)	
Total Length of Stay in Hospital (day)	Yes	12	5.5 ± 3.5	2–12 (4)	0016 *
	No	88	3.18 ± 1.13	2–6 (3)	
Baby Weight (g)	Yes	12	2118.33 ± 956.67	510–3180 (2350)	0.074
	No	88	2631.82 ± 489.92	990–3420 (2675)	
Apgar Score—1st Minute	Yes	12	5.08 ± 3.09	0–9 (6.5)	0.455
	No	88	6.25 ± 1.5	1–9 (6)	
Apgar Score—5th Minute	Yes	12	6.75 ± 3.57	0–10 (8.5)	0.368
	No	88	8.33 ± 1.28	4–10 (9)	
Drain Output on Postoperative Day 1 (mL)	Yes	12	435.83 ± 245	120–1050 (390)	0.240
	No	88	378.35 ± 275.09	100–1600 (300)	

*Mann-Whitney U test* * *p* < 0.05

**Table 4:** Comparison of Measurements According to Complication Status.

		*n*	Mean ± Sd	Min–Max (Median)	*p*
Preoperative Halp Score	Yes	12	24.14 ± 7.36	11.11–31.89 (29.65)	0.023 *
	No	88	22.58 ± 10.53	5.15–66.44 (19.91)	
Postoperative Halp score at 6th hours	Yes	12	21.74 ± 10.48	11.65–38.6 (16.01)	0.013 *
	No	88	23.8 ± 10.38	9.78–61.08 (20.16)	
Postoperative Halp score at 24th hours	Yes	12	22.76 ± 19.96	5.65–60.85 (28.29)	0.065
	No	88	16.56 ± 8.38	3.17–39.49 (15.2)	

*Mann-Whitney U test* * *p* < 0.05

**Table 5:** Determination of Factors Affecting Pre-HALP Measurement—Regression Analysis.

		Univariable					Multivariable				
Model	Variables	B	S. Error	Standard (B)	t	*p*	B	S. Error	Standard (B)	t	*p*
1	Placenta Location	6.868	1.478	0.321	4.648	0.001 **	2.527	1.426	0.118	1.772	0.078
	Complications	−1.563	1.488	−0.076	−1.051	0.295	0.680	1.457	0.033	0.467	0.641
	Age	0.460	0.141	0.231	3.257	0.001 **	0.012	0.117	0.006	0.100	0.921
	Gestational Age at Diagnosis	−0.953	0.093	−0.601	−10.276	0.001 **	−0.603	0.104	−0.380	−5.791	0.001 **
	Gestational Age at Surgery	−1.305	0.164	−0.502	−7.945	0.001 **	−0.359	0.227	−0.138	−1.583	0.115
	Postoperative Hospital Stay	−0.267	0.411	−0.047	−0.651	0.516	−0.061	0.462	−0.011	−0.133	0.895
	Baby Weight	−0.008	0.001	−0.565	−9.385	0.001 **	−0.005	0.001	−0.387	−4.659	0.001 **
	Birth Apgar 1.min	−1.491	0.351	−0.296	−4.247	0.001 **	−1.799	0.658	−0.357	−2.733	0.001 **
	Birth Apgar 5.min	−1.614	0.362	−0.310	−4.465	0.001 **	−2.588	0.762	−0.496	−3.396	0.001 **

** *p* < 0.01

**Table 6:** ROC Analysis Results: Cutoff and AUC.

Parameter	Sensitivity (%)	Specificity (%)	Cutoff Value	Area Under the Curve (AUC)
Pre-HALP	53.2%	82.0%	29.23	0.602

**Table 7:** Bootstrap internal validation results of the HALP score.

Analysis	AUC (Mean, 95% CI)	Cutoff (Mean, 95% CI)	Sensitivity (Mean, 95% CI)	Specificity (Mean, 95% CI)
Bootstrap (1000 resamples, fixed cutoff = 29.23)	0.598 (0.400–0.778)	29.23	0.75 (0.46–1.00)	0.15 (0.08–0.23)
Bootstrap (5000 resamples, optimal cutoff)	0.597 (0.402–0.780)	18.29 (11.78–31.89)	0.71 (0.29–1.00)	0.63 (0.14–0.93)

## Data Availability

The datasets analyzed during the current study are not publicly available due to institutional restrictions but are available from the corresponding author upon reasonable request.
